# A comprehensive analysis of the microbiota composition and gene expression in colorectal cancer

**DOI:** 10.1186/s12866-020-01938-w

**Published:** 2020-10-13

**Authors:** Qian Zhang, Huan Zhao, Dedong Wu, Dayong Cao, Wang Ma

**Affiliations:** 1 Department of Oncology, The First Affiliated Hospital of Zhengzhou University, No. 1 Eastern Jianshe Road, Erqi District, Zhengzhou, 450000 Henan China; 2 Department of Oncology, The First People’s Hospital of Zhengzhou, Zhengzhou, 450004 Henan China; 3 Department of Burns, The First People’s Hospital of Zhengzhou, Zhengzhou, 450004 Henan China

**Keywords:** Colorectal cancer, Gut microflora, Gene expression, Pathways enrichment, Survival analysis

## Abstract

**Background:**

The dysregulation of gut microbiota is pivotal in colorectal carcinogenesis. Meanwhile, altered gut microbiome may affect the development of intestinal diseases through interaction with the host genes. However, the synergy between the altered gut microbiota composition and differential expression of specific genes in colorectal cancer (CRC) remains elusive. Thus, we integrated the data from 16S rRNA gene sequences and RNA sequences to investigate the potential relationship between genes and gut microbes in patients with CRC.

**Results:**

Compared with normal samples, the presence of *Proteobacteria* and *Fusobacteria* increased considerably in CRC samples; conversely, the abundance of *Firmicutes* and *Spirochaetes* decreased markedly. In particular, the genera *Fusobacterium*, *Catenibacterium*, and *Shewanella* were only detected in tumor samples. Meanwhile, a closely interaction between *Butyricimonas* and *Clostridium* was observed in the microbiome network. Furthermore, a total of 246 (differentially expressed genes) DEGs were identified between tumor and normal tissues. Both DEGs and microbiota were involved in bile secretion and steroid hormone biosynthesis pathways. Finally, genes like cytochrome P450 family 3 subfamily A member 4 (*CYP3A4*) and ATP binding cassette subfamily G member 2 (*ABCG2*) enriched in these two pathways were connected with the prognosis of CRC, and CRC patients with low expression level of *CYP3A4* and *ABCG2* had longer survival time.

**Conclusion:**

Identifying the complicated interaction between gut microbiota and the DEGs contributed to further understand the pathogenesis of CRC, and these findings might enable better diagnosis and treatment of CRC patients.

## Background

Colorectal cancer (CRC) is one of the primary causes of mortality and morbidity worldwide, thus representing a major public health issue [1]. Although heritable genetic mutations are closely linked to some types of CRC [2], increasing evidences indicate that diet is regarded as a notable risk factor of CRC [3, 4]. Chan et al. revealed that excessive intake of red meat and animal fat might increase the risk of CRC [5]. It is reported that diet can modulate the composition of gut microbiota which serves a crucial role in maintaining intestinal homeostasis and is involved in the regulation of host inflammation and immune responses [6]. Different members of the intestinal microbiota can jointly regulate the host immune and metabolic systems, subsequently producing carcinogenic or anticancer substances [7, 8]. Lately, accumulating studies have reported the role of intestinal microbiota in health and disease [9, 10]. Flemer et al. proposed that the disharmony of intestinal microbiota might influence the pathogenesis of CRC [11]. Hence, applying strategies to manage the composition of gut microbiota to promote recovery of a favorable microbiota community may be feasible in the treatment of patients with CRC.

Additionally, it has been revealed that an altered gut microbiome may affect the development of intestinal diseases through interaction with the innate immune system and other host genes [12]. Huang and colleagues demonstrated that the possible pathogenic flora of colitis-related cancer was connected with the C-X-C motif receptor 2 (CXCR2) signaling axis during cancer progression [13]. Imhann et al. showed that the interaction between host genetics and intestinal microbiota was the basis of the occurrence and clinical manifestations of inflammatory bowel disease [14]. Moreover, enteric microbiota dysbiosis and genetic abnormalities led to disruption of the intestinal barrier, thus triggering early kidney injury in mice [15]. Furthermore, a large number of studies have shown that dysbiosis of microbiota contributes to cancer susceptibility by affecting multiple pathways. A previous study indicated that gut microbes induced epithelial-to-mesenchymal transitions through various signaling pathways, such as Wnt- and TGF β-signaling pathway, resulting in invasion and metastasis of CRC cells [16]. These findings emphasize that these specific pathways can influence the development of cancer through altering gene expression and microbiota composition [17]. However, pathways that involved in the altered microbiota composition and differential gene expression in CRC have not been well identified. Meanwhile, specific genes that may disrupt the gut microbial composition and ultimately cause CRC remain not well recognized.

With the development of biological information technology, high-throughput sequencing has been widely employed to investigate the pathogenesis of cancer. Meanwhile, multi-omics (metagenomics, transcriptomics, and proteomics) are rapidly expanding our knowledge of the gut microbiota in health and disease. Thompson et al. explored the correlation between the gut bacterial groups and host genes expression in patients with breast cancer by using RNA sequencing data and 16S ribosomal sequencing data [18]. However, a global investigation of the association of tumor gene expression with tumor metagenomics in CRC has not been well described. The obtainment, analysis, and comparison of multi-omics data are challenging tasks. Some actual difficulties must be considered. For example, samples collection, as well as RNA and DNA extraction are challenging; in addition, sequencing costs and run times of metagenomics and transcriptomics analyses are substantially higher and longer than single sequencing analysis. Thus, we used the sequencing data from public databases for analysis. Normally, multi-omics studies should analyze the sequencing results from tumors and matched normal tissues that were from same patients [19, 20]. Unfortunately, analyzing the sequencing data of the same patients were difficult due to our data came from two databases. Therefore, we made an initial exploration of the relationship between microbial composition and gene expression. In the present study, the data of 16S rRNA sequencing and mRNA sequencing were downloaded, followed by identification of the significantly altered gut microbiota and differentially expressed genes (DEGs) between CRC sample and normal sample. In addition, Kyoto Encyclopedia of Genes and Genomes (KEGG) enrichment pathway analysis of differential OUT and DEGs was respectively performed, followed by integration to identify the co-enrichment pathways. Finally, survival analysis of DEGs involved in co-enrichment pathways was further conducted. This study described the relationship between intestinal flora and gene expression in patients with CRC, and provided valuable information for diagnosis and treatment of CRC.

## Results

### Rarefaction curve and diversity analysis

**Fig. 1:**
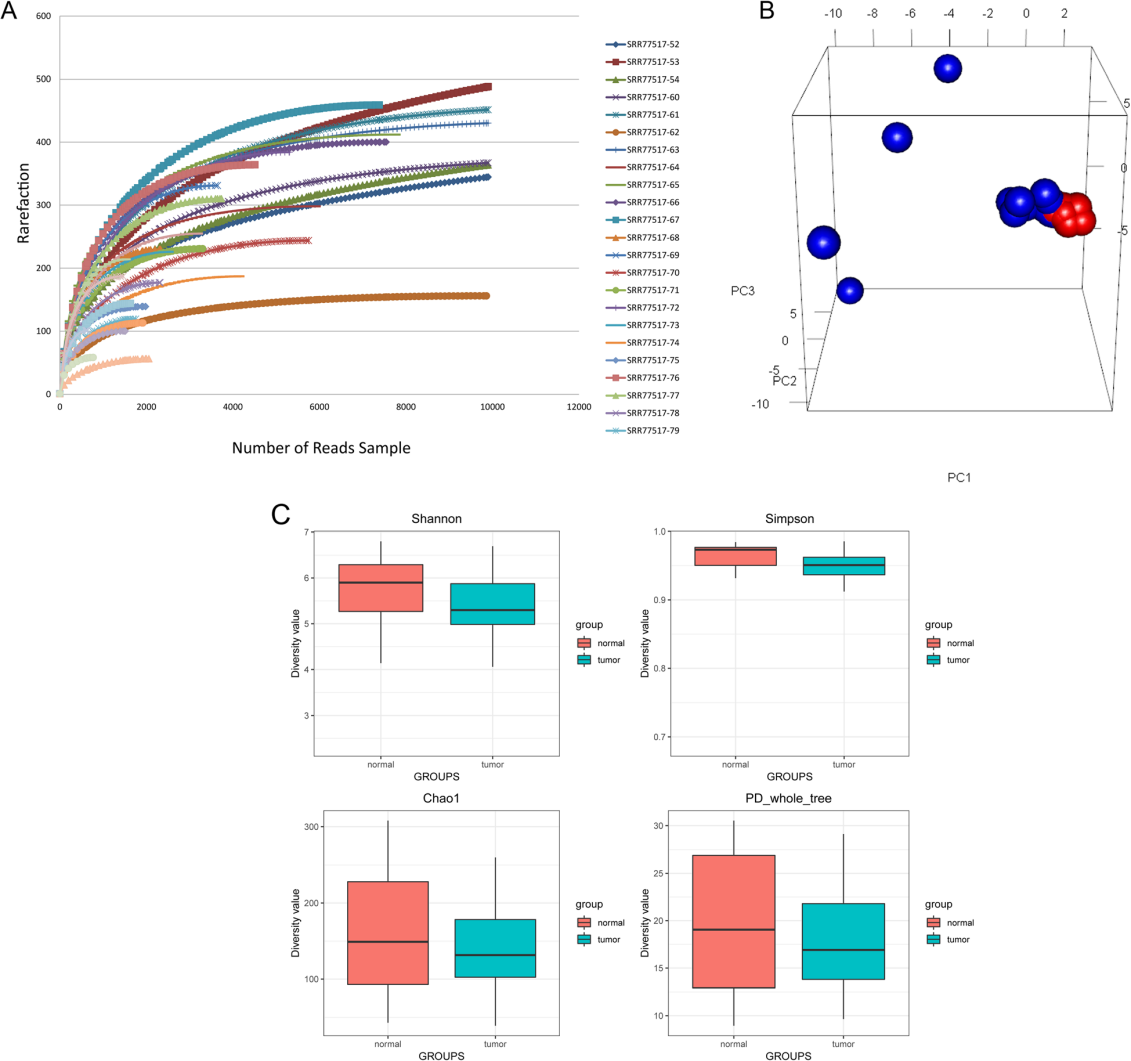
Alpha and beta diversity of CRC and normal samples. (**a** Rarefaction curves of all samples sequenced, indicating the number of OTUs observed with different sequencing depths. **b** PCA plot. **c** Boxplots showing alpha diversity in CRC and normal samples using different metrics (Shannon, Simpson, Chao1, and PD_whole_tree indices))

Rarefaction curves of most samples tend to be flat, suggesting that the amount and depth of sequencing data were reasonable (Fig. 1a). The principal component analysis (PCA) plot showed differences in the gut microbiota composition between CRC and normal samples (Fig. 1b). In addition, we calculated the alpha diversity indices to estimate the diversity of gut microbiota. In the normal samples, the alpha diversity indices were 5.4, 0.9, 149.4, and 18.6 for Shannon, Simpson, chao1, and PD_whole_tree, while these indices for tumor samples were 5.5, 0.9, 147.7, and 18.5. No differences were observed among these four indicators between the two groups. Thus, the alpha diversity results revealed that there was no significant difference between the normal samples and tumor samples (Fig. 1c).

### Taxonomic composition

**Fig. 2:**
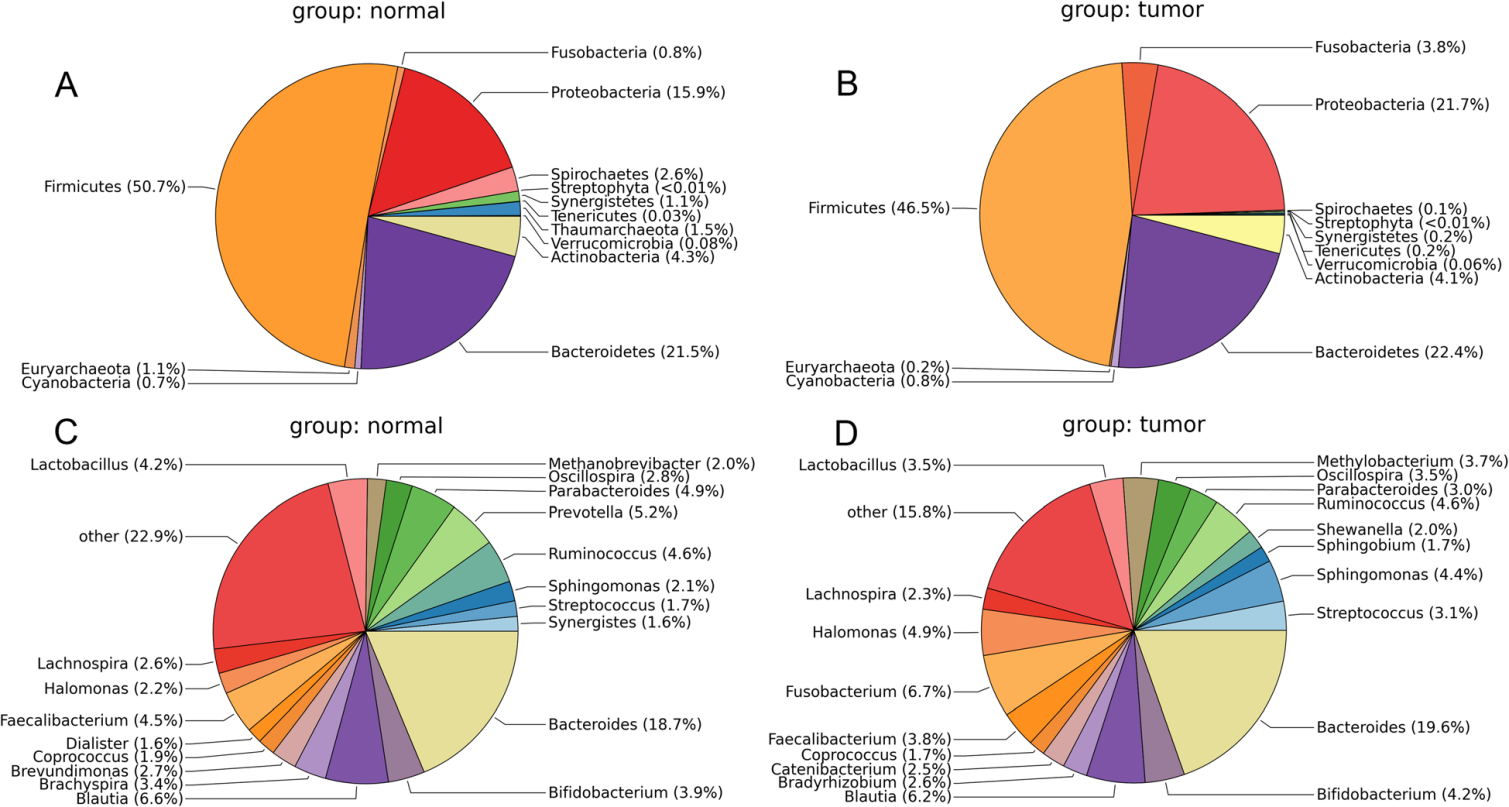
Differential microbiota distribution at phylum (**a**, **b**) and genus (**c**, **d**) level between normal and tumor samples

A total of 13 different phyla were detected from these two groups. The results are shown in Fig. 2a and b. At the phylum level, the microbiota of the tumor and control samples shared 12 phyla, and the members of *Thaumarchaeota* were only identified in the control group. In addition, four dominant phyla were detected among all the samples, including *Firmicutes* (50.7% in normal group and 46.5% in tumor group), *Proteobacteria* (15.9% in normal group and 21.7% in tumor group), *Bacteriodetes* (21.5% in normal group and 22.4% in tumor group), and *Actinobacteria* (4.3% in normal group and 4.1% in tumor group). Compared with the normal group, the relative abundance of *Firmicutes*, *Spirochaetes*, and *Euryarchaeota* in tumor group was decreased, while the abundance of *Fusobacteria*, *Proteobacteria*, and *Bacteroidetes* was increased. Meanwhile, the gut microbiota of the two groups shared 24 genera. The results demonstrated that the level of *Bacteroides* was advantaged across two groups (Fig. 2c and d). Specifically, four dominant genera, including *Bacteroides* (18.7%), *Blautia* (6.6%), *Prevotella* (5.2%), and *Parabacteroides* (4.9%), were observed in normal group. Meanwhile, the abundance of *Bacteroides* (19.6%), *Fusobacterium* (6.7%), and *Blautia* (6.2%) was higher in the tumor group compared with normal group. Notably, *Fusobacterium* (6.7%), *Catenibacterium* (2.5%), and *Shewanella* (2.0%) were specifically detected in the tumor samples.

### Differentially enriched operational taxonomic units (OTU) and pathway enrichment analysis

**Fig. 3:**
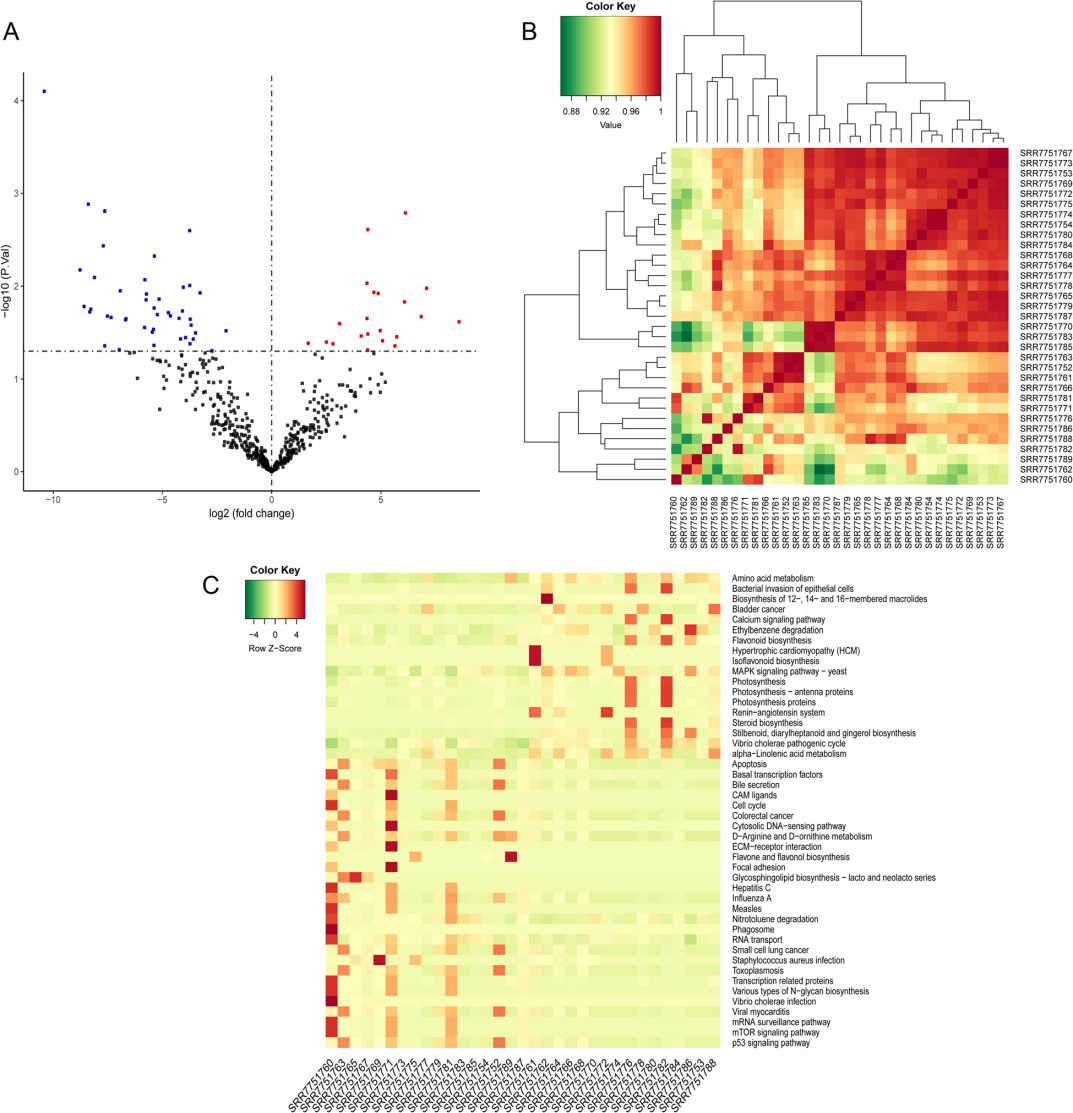
Identification of the different OTUs and KEGG pathway enrichment. (**a** The volcano plot of different OTUs. **b** The heat map of different OTUs. Green represents low expression, and red indicates high expression. **c** KEGG pathways enrichment analysis. Red refers to high expression, while green refers to low expression)

A total of 66 differentially enriched OTUs were identified between CRC samples and normal samples, including 22 up-regulated and 44 down-regulated OTUs. The differential OTUs were visualized by using volcano plot and hierarchical clustering (Fig. 3a and b). There was significant difference between tumor and normal samples. The results indicated that the abundance of A*ctinobacteria* and *Fusobacteria* in tumor samples was significantly higher than that in normal samples. Compared with normal samples, the relative abundance of *Firmicutes* and *Proteobacteria* was observably lower in tumor samples. Meanwhile, the differential OTUs were significantly involved in 67 pathways (Fig. 3c). The results implicated that these OTUs mainly participated in CRC, MAPK signaling pathway, and p53 signaling pathway.

### Network analysis of microbiome

**Fig. 4:**
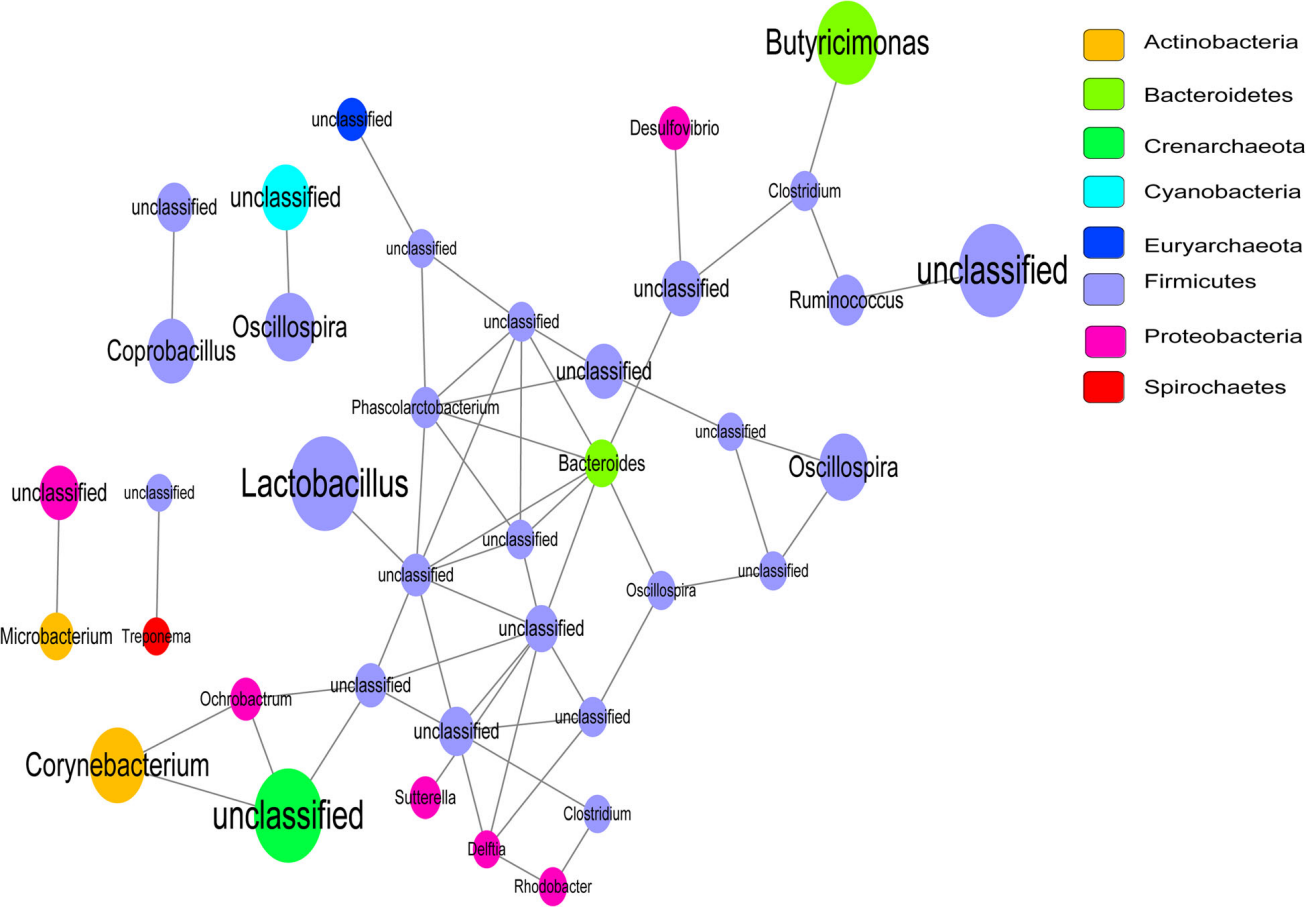
Networks of the bacterial OTUs. Nodes correspond to OTUs and node size corresponds to their relative abundance

Network analysis of differential OTUs to reveal the relationship among microbes. The network composed of 38 nodes and 55 edges was constructed to describe the complex relationships of microbiome (Fig. 4). The 35 genera were from eight bacterial phyla, including 24 genera from *Firmicutes* (57.55%), one genus from *Crenarchaeota* (10.40%), two genera from *Bacteroidetes* (10.39%), two genera from *Actinobacteria* (8.87%), six genera from *Proteobacteria* (6.52%), one genus from *Cyanobacteria* (5.21%), one genus from *Euryarchaeota* (1.03%), and one genus from *Spirochaetes* (0.03%). Bacteria from *Bacteroides*, *Phascolarctobacterium*, and *Delftia* showed interaction with seven, six, and four genera, respectively. Specially, for the genera related to CRC, *Butyricimonas* showed closely connection with *Clostridium*.

### DEG identification and pathway enrichment analysis

**Fig. 5:**
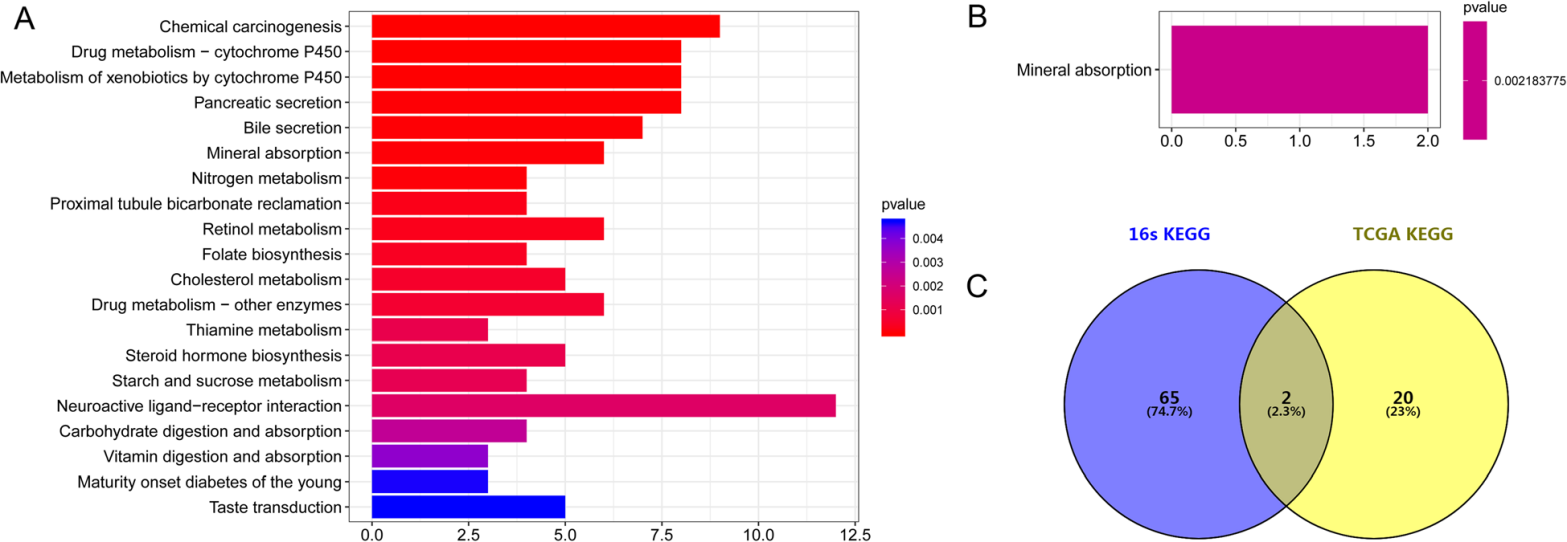
KEGG pathways enrichment analysis. (**a** KEGG pathways analysis of DEGs up-regulated in CRC. **b** KEGG pathways analysis of down-regulated DEGs. **c** Venn diagrams of the KEGG pathways between the different OTUs and DEGs)

A total of 246 DEGs (222 up-regulated and 24 down-regulated genes) were screened between tumor and normal samples. By comparing with the normal group, we found that apolipoprotein B (*APOB*) and carbonic anhydrase 1 (*CA1*) were significantly up-regulated, whereas angiopoietin like 5 (*ANGPTL5*) and shisa family member 7 (*SHISA7*) were down-regulated in the tumor samples. Furthermore, up-regulated DEGs were significantly enriched in 22 KEGG pathways (Fig. 5a), such as genes involved in chemical carcinogenesis, drug metabolism-cytochrome P450, and bile secretion. The down-regulated DEGs were closely involved in mineral absorption (Fig. 5b).

### Integrated analysis

We integrated the pathways enriched by DEGs and OTUs, two overlapping pathways were obtained, namely, bile secretion and steroid hormone biosynthesis. These two pathways could affect CRC not only at transcriptome levels, but also at the intestinal microbiota level (Fig. 5c). Additionally, a total of 11 up-regulated DEGs, including aquaporin 8 (*AQP8*), carbonic anhydrase 2 (*CA2*), solute carrier family 4 member 4 (*SLC4A4*), ATP binding cassette subfamily G member 2 (*ABCG2*), cytochrome P450 family 3 subfamily A member 4 (*CYP3A4*), ATPase Na+/K+ Transporting subunit alpha 2 (*ATP1A2*), ATP binding cassette subfamily B Member 11 (*ABCB11*), hydroxy-delta-5-steroid dehydrogenase, 3 beta- And steroid delta-isomerase 2 (*HSD3B2*), UDP glucuronosyltransferase family 1 member A8 (*UGT1A8*), UDP glucuronosyltransferase family 2 member B10 (*UGT2B10*), and UDP glucuronosyltransferase family 1 member A3 (*UGT1A3*), were significantly involved in these two pathways.

### Survival analysis

**Fig. 6:**
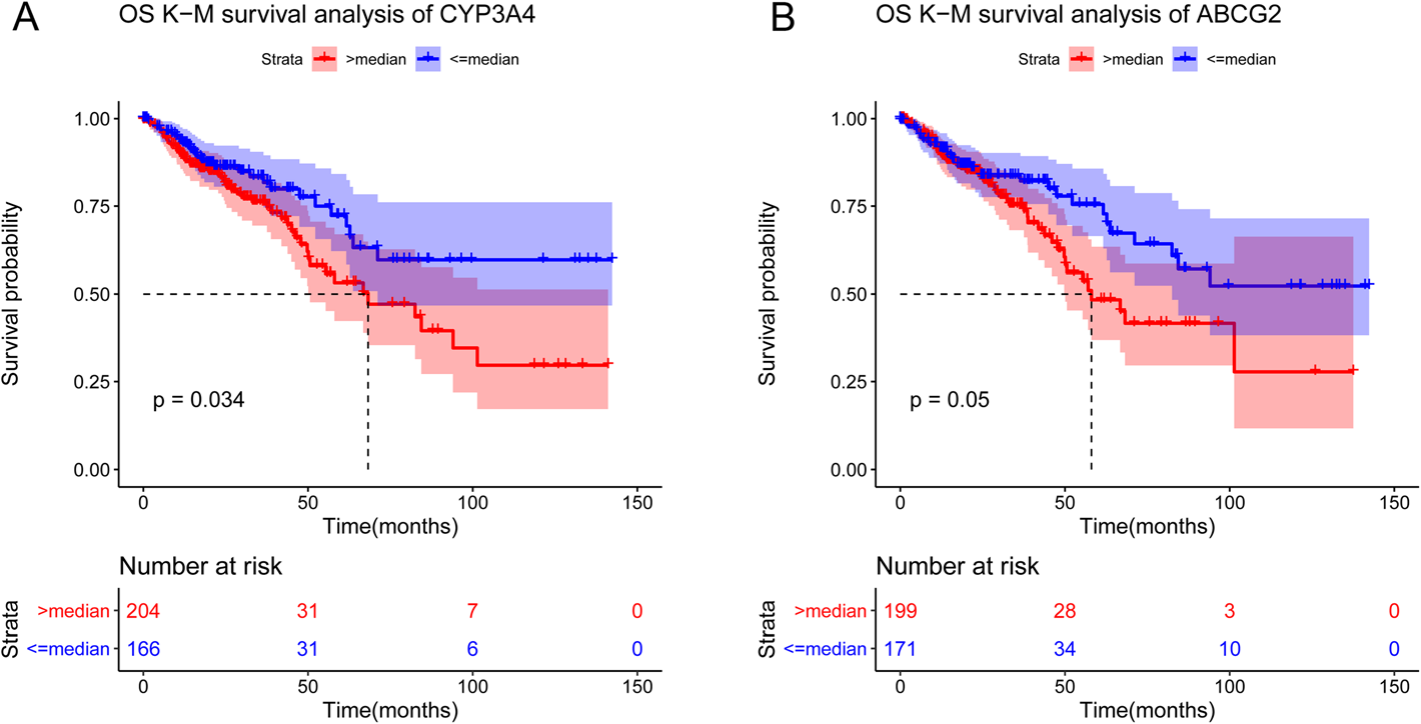
The Kaplan-Meier curves for *CYP3A4* (**a**) and *ABCG2* (**b**)

Survival analysis of above DEGs was performed using Kaplan-Meier (K-M) method. Among these candidate genes, three genes, including *SLC4A4*, *CYP3A4*, and *ABCG2*, were significantly related to the prognostic of CRC. As shown in Fig. 6, CRC patients with low expression level of *CYP3A4* and *ABCG2* had longer survival time.

## Discussion

Genes are known to regulate the pathogenesis of CRC and are associated with the survival outcomes of patients. In addition, host genes can also regulate the growth of microbiota, influencing the composition of the intestinal microbial community [21]. Thus, we investigated the association between mRNA expression and microbiome composition in CRC tissues. In this study, we found that the abundance of *Proteobacteria* and *Fusobacteria* increased in the tumor samples, whereas that of *Firmicutes* decreased. *Fusobacterium*, *Catenibacterium*, and *Shewanella* were specifically detected in the tumor tissues. Additionally, two co-enrichment pathways, including bile secretion and steroid hormone biosynthesis, were associated with both DEGs and differential OTU in CRC. Furthermore, CRC patients with low expression level of *CYP3A4* and *ABCG2* had longer survival time.

The tumor microenvironment of CRC is a complex community of cancer cells, noncancerous cells, and diverse microbiota [22]. The imbalance of gut microbiota may contribute to carcinogenesis. Aleksandar et al. reported that *Fusobacterium* were enriched in carcinomas, whereas the abundance of *Firmicutes* significantly decreased in tumors [23], which was consistent with our findings. Specifically, *Fusobacterium* was markedly enriched in the tumor tissues. A previous study suggested that *Fusobacterium* expressed the virulence factor FadA and activated the WNT signaling pathways, thus promoting growth of CRC [24]. *Fusobacterium* has been shown to inhibit immune responses of CRC tumors [25]. Meanwhile, *Fusobacterium* species are also known to induce host proinflammatory responses and possess virulence [26]. Our findings were supported by the above reports, and highlighted that the clinical relevance of *Fusobacterium* in the development of CRC should be addressed in further studies. In this study, we found *Shewanella* was another genus particularly enriched in the tumor tissues. *Shewanella* was revealed to cause pulmonary and blood infections [27], and it could raise purulent pericarditis with greenish pericardial effusion [28]. Wang et al. suggested that increased *Shewanella* algae was a biomarker of colorectal adenoma [17]. However, its role in CRC tumor progression was not well-defined, further detailed studies were required to verify our findings. Interestingly, a closely interaction between *Butyricimonas* and *Clostridium* was observed in the microbiome network. Wu et al. confirmed that *Butyricimonas* was only detected in CRC group from mouse model [29]. Meanwhile, *Clostridium* was a risk factor for the development of CRC [30]. A previous study suggested that transplanting *Clostridium symbiosum* to germ-free nutrition-deficient mice might promote protein synthesis in local gut epithelium, which might be considered as potential supporter to the development of carcinogenesis [31]. Additionally, *Clostridium symbiosum* was a promising biomarker for early and noninvasive detection of CRC [32]. Taken together, we hypothesized that this relationship might play a pivotal role in the pathogenesis of CRC, and these two microbiotas could be utilized as predictors of CRC diagnosis.

Two pathways were significantly enriched in both DEGs and OTUs, namely, bile secretion and steroid hormone biosynthesis pathways. Moreover, genes like *CYP3A4* and *ABCG2* were involved in these two pathways and were also associated with the prognosis of CRC. *CYP3A4* encodes a member of the cytochrome P450 superfamily of enzyme [33]. A recent study demonstrated that the genotoxicity of the carcinogen was influenced by cytochrome P450 enzyme system, and *CYP3A* was highly expressed in colonic tissue [34]. Zhang et al. pointed out that *CYP3A4* might be recognized as an anti-cancer target, indicating it could be used as a potential molecular marker for predicting and treating CRC [21]. Additionally, *CYP3A* was involved in the metabolism of carcinogen and was associated with inactivation of anticancer drugs [35]. A prior study implicated that the *CYP3A* mRNA transcripts were present in the human colorectal epithelium and CRC cell lines [36]. Similarly, we also showed that *CYP3A4* served an important role in the development of CRC. Furthermore, a study about the relationship between cytochrome P450 and CRC indicated that several P450s were the independent markers of CRC prognosis [34], which were consistent with our results. Pathway enrichment analysis revealed that *CYP3A4* was involved in steroid hormone biosynthesis pathway. The association between steroid hormone biosynthesis pathway and cancer development has been confirmed. A study on gastric cancer (GC) suggested that steroid hormone biosynthesis pathway and their receptors expressions could be altered by genetic variations, thereby contributing to susceptibility to GC [37]. Steroid hormones also played a central role in the progression of prostate cancer, and conversion of adrenal androgen precursors and other steroid-producing pathways might contribute to tumor progression and resistance to therapy [32]. Therefore, we speculated that *CYP3A4* might serve important role in the pathogenesis of CRC via affecting steroid hormone biosynthesis pathway, as well as it might be regarded as a prognostic biomarker and a therapeutic target for CRC.

*ABCG2* is a member of the superfamily of ATP-binding cassette (ABC) transporter protein, which can induce drug resistance and treatment failure in tumor tissues [38]. Liu et al. suggested that *ABCG2* was highly expressed in CRC and it might be involved in progression and metastasis of advanced malignancy cancer [39], which was in line with our finding. Meanwhile, higher *ABCG2* mRNA expression also represented an unfavorable prognostic factor of esophageal squamous cell carcinoma [40]. Thus, we speculated that *ABCG2* might be regarded as a prognostic marker of CRC. In this analysis, we observed that *ABCG2* was involved in bile secretion pathway. *ABCG2* is a hepatobiliary efflux transporter and is involved in the biliary excretion of sulfate conjugates [41] and troglitazone sulfate of therapeutics [42]. Genes such as urothelial cancer associated 1 (*UCA1*) which participated in bile secretion pathway were overexpressed in hepatocellular carcinoma (HCC) tissues [43]. Additionally, bile acids role as tumor promoters have been confirmed by extensive experiments [44, 45]. From the above, *ABCG2* might play role in the pathogenesis of CRC via bile secretion pathway. Although the mechanism of *ABCG2* in CRC progression remained unclear, the importance of *ABCG2* in CRC should not be underestimated.

Taken together, our study highlighted that changes in gene expression and microbiota composition were linked to the specific pathways. We showed that differential expression of genes might cause the alteration of the bile secretion and steroid hormone biosynthesis in CRC tissues, thereby changing the abundance and composition of intestinal microbiota and eventually might trigger the occurrence of cancer. Our results have to be interpreted in light of some limitation. In this analysis, CRC related data were obtained from two different databases and it was not from matched samples. An integrated study based on multi-omics data from the same patient will be our focus in the future. In addition, our study was based on bioinformatics analyses of the datasets from public databases, and further experimental studies and clinic trial must be conducted to validate and strengthen our results.

## Conclusion

By integrating the results of microbiome and transcriptome, we revealed a potential relationship between the genes and gut microbes in patients with CRC, and gained better insight into the pathogenesis and prognosis of CRC. Our study might provide a new perspective for the diagnosis and treatment of CRC, and these genes and microbiota might serve as potential diagnostic markers and therapeutic targets for CRC.

## Methods

### Data resource

The intestinal microbiota data with the number SRP158779 (http://www.ncbi.nlm.nih.gov/sra/SRP158779) were retrieved from the NCBI Sequence Read Archive (SRA) database. This dataset contained 38 samples from 19 patients, including 19 CRC tumors samples and 19 paired non-neoplastic tissues. DNA was extracted and purified using the QIAgen DNA extraction kit. The library was generated based on the V3-V4 region of the 16S rRNA, and then was sequenced on an Illumina HiSeq 2000 platform by using paired-end sequencing. In addition, the mRNA sequencing data (level 3, raw counts) and clinical characteristics of CRC were downloaded from The Cancer Genome Atlas (TCGA) database (http://firebrowse.org/) for a total of 422 samples (371 CRC tumors and 51 normal samples).

### OTU cluster and taxonomy classification

The raw data were converted to fastq format using fastq-dump software (parameter: split-3). Raw data containing low-quality reads that could affect the results of following analysis. Thus, quality control was carried out to obtain high-quality clean reads. Quantitative Insights Into Microbial Ecology (QIIME) (version 1.4.0) [25] software was employed to perform further analysis. Primarily, the paired-end reads were assigned to samples based on their unique barcodes, and then the amplified primers were excised and chimera sequences were removed. Additionally, the clean reads were used for diversity analysis and taxonomic composition based on the Greengenes database (release 13.5, http://greengenes.secondgenome.com/) [46]. Filtered sequences were clustered into OTUs at 97% similarity using UCLUST (version 1.2.22q, http://www.drive5.com/usearch/) [47]. Thereafter, the sequence with the highest abundance in each OTU was selected as the representative sequence of this OTU. Ultimately, based on the number of sequences included in each OTU, the OTU table abundance in each sample was constructed. In addition, taxonomic assignments of OTUs that reached 97% similarity level were performed using RDP classifier (https://sourceforge.net/projects/rdp-classifier/) [48] by comparing with the Greengene database (Release 13.5, http://greengenes.secondgenome.com/) [49].

### Alpha and beta diversity analysis

Abundance and diversity of microbial communities could be reflected by alpha diversity. The Shannon, Simpson, Chao1, and PD_whole_tree indices were calculated to estimate alpha diversity. Concretely, the Shannon and Simpson indices were used to represent the community diversity, and chao 1 indicated the community richness, as well as PD_whole_tree symbolized the phylogenetic diversity. All the statistical analyses were performed using the R phyloseq package [50]. Additionally, the rarefaction curve was plotted to reveal whether the amount of sequencing data was reasonable. Beta diversity analysis could examine the similarity of community structure among different samples. In the present study, beta diversity was calculated by the QIIME software and cluster analysis was conducted by PCA; thereafter, RGL package in R software was applied to visualize the results.

### Screening of differentially OTU

The OTU data were preprocessed by using trimmed mean of M values (TMM) method from edgeR package in R software [51]. Subsequently, the differential analysis was carried out using the quasi-likelihood (QL) F-test from edgeR package. *P* value < 0.05 was considered as statistically significant.

### Network analysis of microbiome

In order to further explore the relationship among differential OTUs, a reciprocal interaction network among microbiome was constructed. Based on the differential OTUs, the correlation coefficient matrix between OTUs was calculated by using R with igraph (version 1.2.2) and psych (version 1.8.4) packages. The pairs with both *p* value < 0.05 and |r| > 0.6 were selected to construct network, and was visualized by using Cytoscape (version 2.8).

### Prediction of the function of differential OTUs

Phylogenetic investigation of communities by reconstruction of unobserved states (PICRUSt) is a computational approach to predict the function of bacteria according to the obtained 16S rRNA gene sequences. In this study, PICRUSt program was used to predict the functional profile of the microbial communities. The main procedures were displayed as following: 1) based on the full-length sequence of 16S rRNA gene of the measured microbial genome, the genetic functional profiles of their common ancestors of differential OTUs were predicted; 2) the functional profiles of other untested species in the Greengenes 16S rRNA gene full-length sequence database were deduced, and then the genetic function prediction spectrum of the entire lineage of archaea and bacteria domain was constructed; and 3) the composition of the sequenced bacteria was mapped into the KEGG database to predict the metabolic function of the microbiota. EdgeR was used to identify the bacteria associated pathways, and *p* value < 0.05 was considered as statistically significant.

### Identification of DEGs and pathway enrichment analysis

The raw reads of the TCGA dataset were transformed on the base (count + 1) logarithm for further analysis. Subsequently, the data were normalized and analyzed by edgeR, and DEGs were selected with |logFC| > 1.5 and false discovery rate (FDR) < 0.05.

The pathway enrichment analysis of DEGs was carried out by using clusterProfiler [30] of R package. Thereafter, gene count ≥2 and *p* value < 0.05 were set as the cut-off criterion.

### Integration of amplicon and transcriptome

To explore the relationship between differential OTUs and DEGs, the functional prediction of differential OTUs and DEGs was integrated. The same or similar pathways that shared between the two sets of data were selected, and the obtained pathways were regarded as CRC-related functions affected by intestinal microbiota.

### Survival analysis

The prognosis outcomes of CRC patients, including overall survival (OS) and survival status, were obtained from TCGA database. The survival analysis of genes involved in obtained pathways was performed. All samples were divided into high and low expression groups according to the median expression level of genes. The Kaplan–Meier survival curves were plotted and statistical significance was assessed using the log-rank tests. *P* < 0.05 was set as the cut-off criteria for statistical significance.

## Data Availability

All data generated or analysed during this study are included in this published article.

## Abbreviations

CRC: Colorectal cancer
NCBI: National Center for Biotechnology Information
SRA: Sequence Read Archive
OTUs: Operational taxonomic units
TCGA: The Cancer Genome Atlas
DEGs: Differentially expressed genes
CXCR2: C-X-C motif receptor 2
KEGG: Kyoto Encyclopedia of Genes and Genomes
QIIME: Quantitative Insights Into Microbial Ecology
PCA: Principal component analysis
OS: Overall survival
*APOB*: Apolipoprotein B
*CA1*: Carbonic anhydrase 1
*ANGPTL5*: Angiopoietin like 5
*SHISA7*: Shisa family member 7
*SLC4A4*: Solute carrier family 4 member 4
*CYP3A4*: Cytochrome P450 family 3 subfamily A member 4
*ABCG2*: ATP binding cassette subfamily G member 2
ABC: ATP-binding cassette
